# Thromboembolic Events Secondary to Endoscopic Cyanoacrylate Injection: Can We Foresee Any Red Flags?

**DOI:** 10.1155/2018/1940592

**Published:** 2018-04-03

**Authors:** Yujen Tseng, Lili Ma, Tiancheng Luo, Xiaoqing Zeng, Yichao Wei, Ling Li, Pengju Xu, Shiyao Chen

**Affiliations:** ^1^Department of Gastroenterology, Zhongshan Hospital, Fudan University, Shanghai, China; ^2^Department of Endoscopy Center, Zhongshan Hospital, Fudan University, Shanghai, China; ^3^Department of Geriatrics, Zhongshan Hospital, Fudan University, Shanghai, China; ^4^Department of Radiology, Zhongshan Hospital, Fudan University, Shanghai, China; ^5^Department of Gastroenterology, Endoscopy Center and Evidence-Based Medicine Center, Zhongshan Hospital, Fudan University, Shanghai, China

## Abstract

**Background:**

Gastric varices (GV) are associated with high morbidity and mortality in patients with portal hypertension. Endoscopic cyanoacrylate injection is the first-line recommended therapy for GV obliteration. This study aims to explore the reason behind related adverse events and better prevent its occurrence.

**Methods:**

A retrospective case series study was conducted from January 1, 2013, to December 31, 2016, to identify patients who experienced severe adverse events secondary to endoscopic cyanoacrylate injection. A literature review of similar cases was performed on two medical databases, Medline and Embase.

**Results:**

A total of 652 patients underwent cyanoacrylate injection at our center within the study duration. Five cases of severe adverse events related to the use of tissue adhesives were identified. Detailed clinical presentation, patient treatment, and outcomes were reviewed and analyzed. Twenty-seven similar cases were identified based on the literature review providing further insight into the study.

**Conclusion:**

Although rare in incidence, systemic embolism associated with cyanoacrylate injection is often fatal or debilitating. This report may raise awareness in treatment protocol, including the necessity of preoperative angiographic studies, to avoid similar adverse events in clinical practice.

## 1. Introduction

Variceal hemorrhage is a fatal presentation of portal hypertension, commonly seen in patients with decompensated cirrhosis. Current treatment protocol for gastroesophageal varices includes primary prophylaxis, management of acute bleeding, and secondary prophylaxis [1]. According to the Baveno VI consensus, a combination of nonselective beta blockers (NSBB) and endoscopic variceal ligation (EVL) for esophageal varices and cyanoacrylate injection for gastric varices are recommended as first-line therapy [2]. Compared to esophageal varices, gastric varices are lower in prevalence but are associated with a higher risk of hemorrhage and mortality [3]. The use of* N*-butyl-2-cyanoacrylate (NB2-CYA) for gastric variceal obliteration was first reported in 1986 and is currently well recognized as first-line therapy with a high hemostasis rate [4–6]. Large cohort studies have demonstrated the safety and efficacy of cyanoacrylate injection; however others have highlighted individual adverse events [7–9]. Occurrence of systemic embolization is often associated with patient morbidity and mortality. We hereby report a series of adverse events associated with cyanoacrylate injection for the treatment of gastric varices.

## 2. Methods

A retrospective case series study was conducted at a tertiary hospital. The hospital database was reviewed; approval was granted by the hospital's institutional review board (IRB). All patients who underwent endoscopic procedure had signed informed consent acknowledging the purpose and risk associated with the intervention. We included (1) patients with gastric varices with or without concurrent esophageal varices treated with injection of N-butyl-cyanoacrylate and (2) patients who experienced severe adverse events (SAE) associated with cyanoacrylate injection within 48 hours of the endoscopic procedure. SAE was defined as occurrence of death, life-threatening disability, or permanent deficit, resulting in a prolonged hospital stay.

All endoscopic procedures were commenced after an overnight fast. First, a routine endoscopy exam was performed to assess the extent of gastroesophageal varices that were classified according to Sarin's classification. Concurrent esophageal varices were graded according to the Japanese Society of Portal Hypertension [10]. Each patient received individualized therapy as deemed fit by the operator. Gastric varices were uniformly treated via the sandwich technique, which starts with an injection of lauromacrogol (Tianyu Pharmaceutical, Zhejiang, China), followed by N-butyl cyanoacrylate (Beijing Suncon Medical Adhesive, Beijing, China), and then finished with flush of lauromacrogol [11]. The number of injection sites and volume of lauromacrogol and cyanoacrylate used directly correlated with the size of the varix. Multiple injection sites were chosen in attempt to obliterate the varix or varices in one session. Volume of lauromacrogol used ranged from 2 to 10 ml, while that of cyanoacrylate ranged from 0.5 to 2 ml, per injection site. Concurrent esophageal varices were treated with either endoscopic band ligation (EBL) or endoscopic sclerotherapy injection (EIS) determined by the operator.

Patients were hospitalized for postoperative observations for 24–48 hours. Any occurrence of severe adverse events (SAE), as previously defined, was recorded. Treatment and patient response secondary to the adverse events were documented. Patient follow-ups were accomplished via telephone interviews or out-patient services to determine survival or further complications.

A literature review of case reports on adverse events related to cyanoacrylate injection was also conducted, specifically, occurrence of embolic or infarction events. Detailed search strategy of Medline (R), from 1946 to present with daily updates, and Embase, from 1974 to March 20, 2017, is provided in the Appendix.

## 3. Results

A thorough review of the inpatient and endoscopy database was carried out from January 1, 2013, to December 31, 2016. A total of 652 patients who underwent N-butyl-cyanoacrylate (NBCA) injection as secondary prophylaxis for gastric variceal hemorrhage were identified. Based on the a priori established inclusion criteria, the detailed hospital record and treatment protocol of 5 patients were reviewed for the purpose of this study. Three of the five patients were male, ranging from 50 to 74 years. The cause of cirrhosis was PBC in the two female patients, while the remaining were due to HBV, HCV, or alcohol, respectively. Three patients were classified as Child-Pugh Class A, while the remainder were Child-Pugh Class B. Two of the five patients were admitted to our hospital due to an episode of acute variceal hemorrhage, while others had either achieved hemodynamic stability or were admitted for a follow-up endoscopic examination. Prior to the procedure, two patients (patients (4) and (5)) received a combination of hemostatic agents and somatostatin. None of the patients had concurrent HCC or hepatic encephalopathy. Detailed patient characteristics are summarized in Table 1.

**Figure 1:**
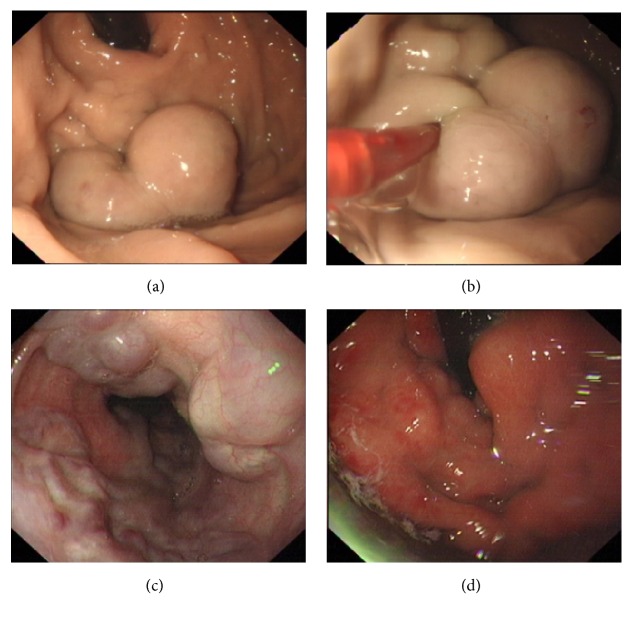
Endoscopic findings of gastroesophageal varices (IGV Type 1 and F3/GOV Type 2) with red wale sign.

Based on the findings of the routine endoscopy, one patient had IGV Type 1, one had GOV Type 1, while three had GOV Type 2 (Figure 1). All gastric varices were treated with the sandwich technique injection of lauromacrogol and cyanoacrylate. The total volume of cyanoacrylate used ranged from 1.0 to 3.5 ml (average 2.7 ml), without exceeding 1.5 ml per injection site. Patients with concurrent esophageal varices were treated with either endoscopic band ligation (EBL) or endoscopic injection sclerotherapy (EIS).

**Figure 2:**
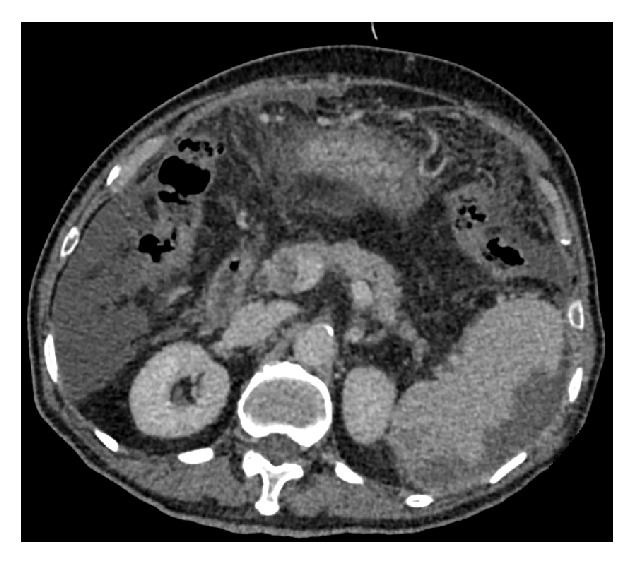
Large area splenic infarct based on CT angiography of the portal venous system.

**Figure 3:**
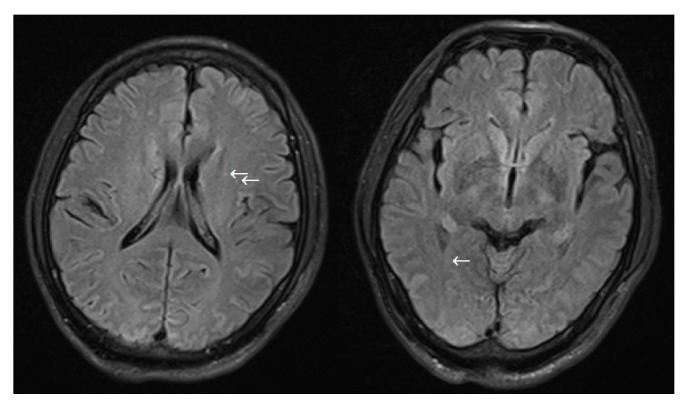
Diffuse hyperdense signals (*←*) on the cerebral MRI, indicative of acute cerebral infarction.

**Figure 4:**
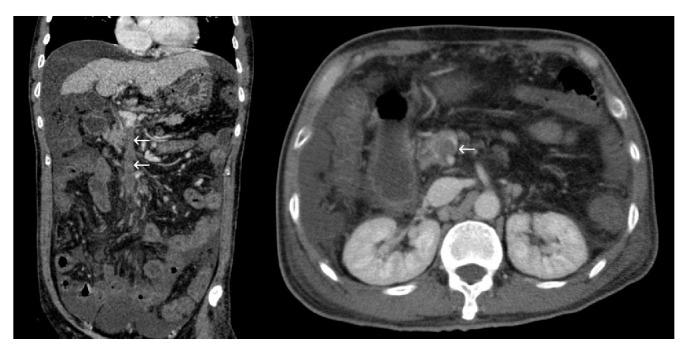
Intraluminal filling defect along the mesenteric vein and edema of the bowel wall (*←*).

One female patient (patient (1)) suffered from cardiac arrest during the procedure. The bedside echocardiogram revealed an enlarged right ventricle and right atrium, widened vena cava, and shrunken left ventricle. Despite aggressive measures including drug and equipment resuscitation, the patient did not survive. Patient (2) experienced fever, severe abdominal pain, and rebound tenderness after the endoscopic procedure due to a large area splenic infarct (Figure 2), confirmed via CTA of the portal venous system. Two patients (patients (3) and (5)) became lethargic and confused and experienced loss of consciousness following endotherapy. Based on clinical symptoms and cerebral MRI findings, both were diagnosed with acute cerebral infarction (Figure 3). The last patient (patient (4)) experienced pain around the umbilical region with a low-grade fever (37.9°C) after the procedure. A subsequent abdominal CT and intestinal mesenteric CTA revealed intraluminal filling defects consistent with acute mesenteric ischemia (Figure 4). Detailed postoperative findings are listed in Table 2.

All patients received hemostatic medication after the endoscopic procedure as part of the standard protocol at our hospital to prevent postoperative hemorrhage (Table 2). Once the patient developed signs of systemic embolization, all hemostatic agents were suspended. All patients were treated with a subcutaneous injection of low-molecular weight heparin (LMWH). Three of the four patients responded well to therapy and were subsequently discharged. Follow-up interviews confirmed survival in all three patients. However, one patient (patient (4)) developed a recurrent GI bleed, presented as melena, after 5 days of anticoagulation treatment. The patient also developed hepatic encephalopathy and deteriorated rapidly. Extraordinary life sustaining measures were refused and the patient died 9 days after the initial procedure. The overall rebleeding rate was 20% and mortality rate was 40% in the five patients who experienced SAE after cyanoacrylate injection. Of the three patients who survived (60%), only 2 received follow-up endoscopy examination. Complete variceal obliteration was observed in one patient (50%), while the other patient had recurrent gastroesophageal varices (GOV Type 2) treated with consolidation EBL plus cyanoacrylate injection.

A retrospective review of the radiological studies was conducted in attempt to identify a potential explanation for the occurrence of an embolic event. Three of the 5 patients had evident spontaneous portosystemic shunts upon review of imaging studies, including one case of portorenal shunt (patient (3), cerebral infarction), one case of portoazygous shunt (patient (5), cerebral infarction), and one case of concurrent portorenal and portosystemic shunt (patient (1), pulmonary embolism). The remaining cases of mesenteric and splenic infarction had no prominent vascular anomaly.

In order to further identify similar reports of adverse events in present literature, a detailed search of Medline (R), from 1946 to present with daily updates, and Embase, from 1974 to March 20, 2017, was conducted (the Appendix). A total of 43 and 119 reports were retrieved from each database, respectively. Forty-two duplicates were removed and a thorough review of title and abstract of 120 articles was performed. Ninety-seven reports were further eliminated due to irrelevance and finally 24 articles, along with 4 case reports identified from other sources, were included for the purpose of this literature review.

Of the 27 studies included, majority of reported adverse events were pulmonary embolism, 12/27 (44.44%), and splenic infarction, 9/27 (33.33%), while others include cases of portal vein, renal vein embolism, sclerosant extravasation, myocardial infarction, diaphragmatic embolism, cerebral infarction, right atrium emboli, esophageal variceal embolism, and subsequent septicemia or DIC. Several adverse events were attributed to cardiac abnormalities such as patent foramen ovale, prompting right-to-left shunt. Other hypotheses include volume and speed of injection or intravariceal pressure, resulting in regurgitation through the portovenous system. Interestingly, many authors presumed the presence of spontaneous portovenous shunt, such as gastrosplenorenal shunt or anomalous arteriovenous shunts, as a culprit for distant embolization. However, none of the reports provided radiological or morphological evidence of the vasculature anomaly. The results of the literature review were summarized in Table 3.

## 4. Discussion

Gastric varices are associated with a high morbidity and mortality rate in patients with portal hypertension. The current recommendation for first-line treatment is endoscopic injection of tissue adhesives. Obliteration can be achieved in one session, but sometimes repeat sessions are required [39]. Although cyanoacrylate injection has proven to be safe and effective, several reports on related adverse events have also been documented [7]. Seewald et al. have emphasized the importance of a standardized technique, which can minimize the risk of embolization and local complications but also decrease variceal recurrence or rebleeding by effectively obliterating vessel tributaries. The recommended mixture proportion of N-butyl-2-cyanoacrylate to lipiodol is 0.5 ml : 0.8 ml, and injection of over 1 ml glue mixture may increase the risk of embolization [8, 40]. Researchers have also explored alternative treatments for gastric varices obliteration, minimizing or eliminating the use of tissue adhesives. Tan et al. conducted a randomized control trial comparing the efficacy of gastric variceal band ligation versus cyanoacrylate injection [41]. Meanwhile, Romero-Castro et al. reported fewer complications with endoscopic ultrasound-guided coil injection compared to that of traditional cyanoacrylate injection [42].

**Figure 5:**
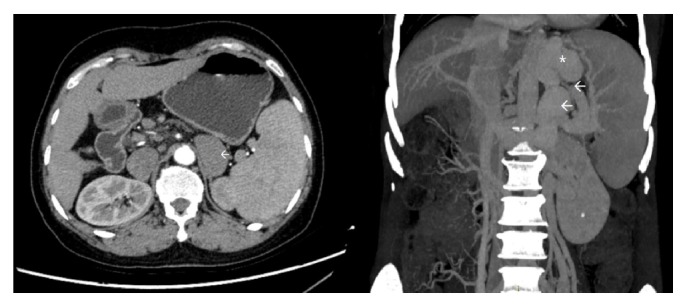
Spontaneous portosystemic shunt in the patient with IGV Type 1, presenting as portorenal and portosystemic shunt (*←*). The coronal view shows gastric varices (*∗*) connected to both the left renal and splenic vein through as large torturous, dilated venous shunt (*←*).

We report five cases of adverse events that occurred after the endoscopic injection of cyanoacrylate for the treatment of gastric varices. All cases involved the formation of systemic embolus, including cerebral vascular infarction, mesenteric infarction, splenic infarction, and pulmonary embolism. A retrospective review of radiological studies revealed presence of spontaneous portosystemic shunt (SPSS) in 3 patients with distant systemic emboli, including one case of portorenal shunt, one case of portoazygous shunt, and one case of concurrent portorenal and portosplenic shunt (Figure 5). Based on the clinical presentation and radiological findings, three cases can be ascertained as glue emboli, including the case of pulmonary embolism and two cases of cerebral infarction. The formation of spontaneous portosystemic shunts (SPSS) may serve as a shortcut for acute glue embolization, which calls into question the necessity of angiographic studies prior to endoscopic intervention and whether patients with diverging shunts should be tackled with a different therapeutic approach [43]. Our center has previously performed BRTO assisted cyanoacrylate injection for patients with large gastrorenal shunt or splenorenal shunt (data reported elsewhere). This procedure prevents the occurrence of systemic glue emboli for patients with evident portosystemic shunt; however, it is poorly tolerated by patients. BRTO assisted cyanoacrylate injection requires the patient to lay in a supine position with only local anesthesia and an angiography of the portosystemic system is performed via femoral access. After the portosystemic shunt is located a balloon is deployed and secured, while the endoscopist performs the subsequent cyanoacrylate injection.

The remaining cases of mesenteric infarction and splenic infarct remain controversial and cannot be ascertained as the presence of SPSS. A plausible explanation could be due to the injection of cyanoacrylate into the arterial system, which in some cases is located adjacent to the varix or is connected via an arteriovenous malformation. Glue emboli of the splenic artery may result in a large area splenic infarct as seen in patient (2). Another explanation is the regurgitation of tissue adhesives through the portovenous system, potentially due to high speed or volume injection or high intravariceal pressure. Patients with end-stage cirrhosis are also prone to clot formation, especially in the portal venous system [44]. The use of various hemostatic agents combined with a decrease in blood flow velocity, exacerbated by a stress event (endotherapy), may also be a probable explanation for an acute thrombus formation. Unlike other studies, our center employs lauromacrogol instead of lipiodol as a diluting agent for cyanoacrylate via sandwich technique [11]. Therefore, glue embolization is difficult to differentiate from a thrombus formation on imaging studies.

Antithrombotic treatment with LMWH is a fairly standard treatment protocol. However, in cases with recent interventional procedure or hemorrhagic episode, the use of LMWH can be precarious [45]. Development of a rebleed in such patients can be just as fatal as the adverse event itself. Anticoagulants are effective in the treatment of blood thrombus; however, the effect on glue emboli or improvement of patient outcome remains questionable.

The detailed literature review provided some further insights based on case reports of embolic events experienced after cyanoacrylate injection. Many authors theorized the presence of spontaneous portosystemic shunt as a probable explanation for embolization of tissue adhesives. However, no radiological or morphological evidence of vasculature malformation was provided. In our study, we meticulously reviewed the radiological imaging of all 5 patients and were able to identify the presence of spontaneous portosystemic shunt in 3/5 (60%) subjects.

Overall, the use of cyanoacrylate for gastric variceal obliteration is widely accepted with promising results. The safety of tissue adhesive injection is often guaranteed when endoscopist abides by the standardized sandwich technique [8, 40]. However, the necessity of preoperative imaging of the portovenous system should also be considered to identify patients with spontaneous portosystemic shunt (SPSS). In such cases, the risk of traditional endoscopic glue injection should be thoroughly vetted, or alternative treatment measures such as coil injection, TIPS, BRTO, or surgical therapy should be referred to. Utility of pre- and postoperative hemostatic agents should also be carefully considered to achieve a desirable hemostatic balance. Adverse events associated with tissue adhesives are often fatal and debilitating for patients; any red flags before endoscopic therapy should be well recognized by physicians, prompting well-rounded consideration to effectively avoid the occurrence of adverse events.

## Figures and Tables

**Table 1:** Summary of patient characteristics, preoperative management, endoscopic findings, and subsequent treatment.

Patient	Cause of PH	Child-Pugh Class	Acute bleed	Preoperative drug	Endoscopic findings	Endoscopic treatment	Volume of cyanoacrylate
(1) 57 y/F	PBC	A	No	None	F0/IGV 1	NBCA	3.5 ml

(2) 74 y/M	Alcohol	A	No	None	F2/GOV2	NBCA + EIS	3 ml

(3) 50 y/M	HCV	A	No	None	F3/GOV2	NBCA + EBL	3.5 ml

(4) 51 y/M	HBV	B	Yes	Aminomethylbenzoic acid 0.4 g Etamsylate 2 g Carbazochrome 80 mg Hemocoagulase 1 IU Somatostatin 6 mg	F3/GOV2	NBCA + EBL	2.5 ml

(5) 52 y/F	PBC	B	Yes	Carbazochrome 80 mg Hemocoagulase 1 IU Somatostatin 6 mg	F3/GOV2	NBCA + EBL	1 ml

**Table 2:** Postoperative events including subsequent severe adverse event (SAE), patient outcome, and probable cause.

Patient	Postoperative drug use	Adverse event	Treatment	Hospital stay	Outcome	Probable cause
(1)	None	Acute pulmonary embolism	BCLS	1 day	Death	Large spontaneous gastrorenal and splenorenal shunt

(2)	Carbazochrome 80 mg Vitamin K1 10 mg Somatostatin 6 mg	Acute splenic infarction	Dalteparin 5000 IU Antibiotics (meropenem + vancomycin)	64 days	Survival	Regurgitation of tissue adhesive through the portovenous system or probable AVM

(3)	Carbazochrome 80 mg Somatostatin 3 mg Hemocoagulase 2 U	Acute cerebral infarction	Dalteparin 5000 IU Edaravone Mannitol Dexamethasone	13 days	Survival	Spontaneous portorenal shunt

(4)	Hemocoagulase 1 IU Somatostatin 6 mg	Acute superior mesenteric infarction	LMWH 4000 IU Simethicone p.o.	9 days	Death	Regurgitation of tissue adhesive through the portovenous system or probable AVM

(5)	Hemocoagulase 1 IU Somatostatin 6 mg	Acute cerebral infarction	LMWH 4000 IU Citicoline GM-1 Dexamethasone	42 days	Survival	Spontaneous portoazygous shunt

**Table 3:** Summary of adverse events related to cyanoacrylate injection found in current literature.

Study	Year	Country	Patient	Glue mixture (ratio), volume	Adverse event	Treatment	Outcome	Probable cause
Shim et al. [12]	1996	S. Korea	59 y/M	(0.5 : 0.8) 7 ml + 2 ml	Portal and splenic vein thrombosis	NA	NA	Large volume injection

Battaglia et al. [13]	2000	Italy	65 y/F	(1 : 1) 6 ml	Intraparenchymal subcapsular hematoma of the spleen	Splenectomy	Survival	Resin occluded branches of splenic vascularization or embolized intraparenchymal vessels and had been eliminated by macrophage action

Türler et al. [14]	2001	Germany	18 y/M	(1 : 1) 5 ml + 2 ml	Pulmonary embolism and left renal vein; recurrent left kidney abscess (5 months)	Thrombectomy and ventilation support; operative and CT-guided drainage (kidney abscess)	Survival	Spontaneous splenorenal shunt

Tan et al. [15]	2002	Malaysia	53 y/M	(0.5 : 0.7) 6 ml +1 ml	Pulmonary and splenic infarction	Supportive treatment and antibiotics	Survival	Collateral portosystemic circulation and presumable anomalous arteriovenous pulmonary shunts

Cheng et al. [16]	2004	Taiwan	65 y/F	(1 : 1) 3 ml	Sclerosant extravasation	Antibiotics and supportive treatment	Survival	High intravariceal pressure and large volume or high injection speed of tissue adhesive

Rickman et al. [17]	2004	USA	55 y/M	(1 : 1) 4 ml + 2 ml	Pulmonary emboli, splenic infarction	Oxygen support	Survival	NA

Kok et al. [18]	2004	S. Africa	24 y/F	(1 : 1) 2 ml + (1 : 2) 5 ml	Pulmonary infarction and septicemia	TIPS surgery	Death	Collateral vessels, size of varices, volume of injection, dilution of lipiodol

Upadhyay et al. [19]	2005	Oman	65 y/M	(1.5 : 2.1)	Inferior wall myocardial infarction and cortical blindness	Percutaneous occlusion of PFO followed by TIPS surgery	Survival	Patent foramen ovale

Alexander et al. [20]	2006	Australia	52 y/M	(1 : 3) 4 ml + 2 ml	Pulmonary embolism	Prednisolone and supportive treatment	Survival	Large volume injection

Liu et al. [21]	2006	Taiwan	42 y/M	(1 : 1) 2 ml	Splenic vein thrombosis	Antibiotics and supportive treatment	Survival	Volume of injection

Martins Santos et al. [22]	2007	Brazil	53 y/M	(1 : 1) 1 ml	Splenic infarction	Antibiotics and supportive treatment	Death	Arteriovenous shunt (probable)

Yu et al. [23]	2007	Taiwan	57 y/M	(1 : 0.7) 1.7 ml	Diaphragmatic embolism	Supportive treatment with narcotic analgesic and short course of terlipressin	Survival	Portophrenic shunt (probable)

Chang et al. [24]	2008	Taiwan	53 y/M	(1 : 1) 2 ml	Pyogenic Portal venous thrombosis	Antibiotics	Death	Direct injection or regurgitation of tissue adhesive along the short gastric vein and splenic vein into the portal vein

Marion-Audibert et al. [25]	2008	France	77 y	(1 : 1) 1.5 ml	Pulmonary embolism	BCLS protocol	Death	Portosystemic vascular shunt (gastrosplenorenal shunt) (reconstruction with animal model)

Abdullah et al. [26]	2009	Malaysia	40 y/M	(1 : 1) 3 ml	Cerebral infarction	NA	Survival	Patent foramen ovale

Park et al. [27]	2010	S. Korea	34 y/M	NA	Right atrium emboli extended from inferior vena cava	NA	Survival	Gastrorenal shunt

Chen et al. [28]	2011	Taiwan	57 y/F	(1 : 1) 4 ml + 6 ml	Esophageal variceal embolism	Cyanoacrylate hemostasis	Survival	NA

Kazi et al. [29]	2012	Australia	44 y/F	(1 : 1) 4 ml	Pulmonary emboli and pulmonary infarct, resulting in DIC	Blood transfusion to correct coagulopathy	Survival	NA

Miyakoda et al. [30]	2012	Japan	76 y/F	(1 : 0.5) 1.5 ml + (1 : 0.5) 3.5 ml	Right atrium emboli	Heparin	Death	NA

Chan et al. [31]	2012	Malaysia	44 y/F	(0.5 : 0.8) 1.3 ml	Splenic infarction	Conservative treatment (analgesics, antihistamine, and antiemetic)	Survival	NA

Singer et al. [32]	2012	USA	75 y/M	(1 : 1) 3 ml	Pulmonary infarction	Empiric antibiotics and supportive care	Survival	NA

Mourin et al. [33]	2012	France	69 y/M	NA	Pulmonary infarction	Anticoagulant treatment + pneumonectomy	Death	Presumed portosystemic vascular shunts

Köksal et al. [34]	2013	Turkey	33 y/F	(1 : 1) 2 ml	Splenic infarction	Supportive treatment	Survival	Retrograde embolization through the splenic vein

Myung et al. [35]	2013	S. Korea	55 y/F	(1 : 1) 2 ml	Splenic infarction and cerebral infarction	NA	Survival	Patent foramen ovale

Nawrot et al. [36]	2014	Poland	54 y/F	(0.5 : 0.8) 12 ml	Pulmonary embolism with septicemia	Antibiotics	Survival	Large spontaneous splenorenal shunt

Chew et al. [37]	2014	UK	34 y/M	(1 : 1) 4 ml	Pulmonary emboli	Intravenous diuretics, empiric antibiotics	Survival	NA

Burke et al. [38]	2017	Australia	25 y/F	1 ml + 3 ml	Pulmonary emboli	BCLA protocol	Death	Presumed collateral circulation

**Table 4:** 

Number	Searches	Medline results	Embase results	Search type
(1)	(esophag^*∗*^ or esophag^*∗*^ gastr^*∗*^ or gastr^*∗*^ esophag^*∗*^ or gastr^*∗*^ oesophag^*∗*^ or gastroesophag^*∗*^ or gastrooesophag^*∗*^ or oesophag^*∗*^ or oesophag^*∗*^ gastr^*∗*^ or gastr^*∗*^).mp. [mp = title, abstract, original title, name of substance word, subject heading word, keyword heading word, protocol supplementary concept word, rare disease supplementary concept word, unique identifier, synonyms]	169513	268529	Advanced

(2)	1 and (varic^*∗*^ or varix).mp.	14601	21427	Advanced

(3)	exp esophageal varices/	12569	17997	Advanced

(4)	exp gastric varices/	12569	2864	Advanced

(5)	(3) or (4)	12569	19501	Advanced

(6)	(2) or (5)	14601	22623	Advanced

(7)	(cyanoacrylate or n-butyl-2-cyanoacrylate or NBCA or NB2CYA or NB2-CYA or tissue adhesive or tissue glue or glue).mp. [mp = title, abstract, original title, name of substance word, subject heading word, keyword heading word, protocol supplementary concept word, rare disease supplementary concept word, unique identifier, synonyms]	13485	26245	Advanced

(8)	(infarct^*∗*^ or embol^*∗*^ or advers^*∗*^ event^*∗*^ or severe advers^*∗*^ event^*∗*^ or complicat^*∗*^).mp. [mp = title, abstract, original title, name of substance word, subject heading word, keyword heading word, protocol supplementary concept word, rare disease supplementary concept word, unique identifier, synonyms]	1649685	3380532	Advanced

(9)	(7) and (8)	4356	10406	Advanced

(10)	(endoscop^*∗*^ therap^*∗*^ or endoscop^*∗*^ treat^*∗*^ or endoscop^*∗*^ inject^*∗*^).mp. [mp = title, abstract, original title, name of substance word, subject heading word, keyword heading word, protocol supplementary concept word, rare disease supplementary concept word, unique identifier, synonyms]	8912	21219	Advanced

(11)	(9) and (10)	186	657	Advanced

(12)	(case or case report^*∗*^ or case serie^*∗*^ or report^*∗*^).mp. [mp = title, abstract, original title, name of substance word, subject heading word, keyword heading word, protocol supplementary concept word, rare disease supplementary concept word, unique identifier, synonyms]	4746115	6342357	Advanced

(13)	(6) and (11) and (12)	43	119	Advanced

Exp, explode.

## Appendix

### Detailed Search Strategy

The search strategy used was Ovid Medline (R), from 1946 to present with daily updates, and Embase, from 1974 to March 20, 2017(see Table 4).
